# Supplementary material for: Nonlinear effects of intrinsic dynamics on temporal encoding in a model of avian auditory cortex
Source: PLoS Comput Biol. 2021 Feb 22;17(2):e1008768. doi: 10.1371/journal.pcbi.1008768 (PMC7932506; doi:10.1371/journal.pcbi.1008768)
Supplement: S1 Text — The six figures have are in the same order as the examples in Fig 4 and have the same format as each other: (a) Left, input RF in the LDC model. Right, estimated RF from GLM. A novel birdsong stimulus was used to compare GLM performance to output of LDC model, with 5s of the spectrogram shown in (b). Voltage traces of LDC model in response to stimulus for a single trial are shown in (c) with corresponding KLT current (d). Spike trains for all 50 trials are shown in (e), with black corresponding to the LDC model and red to the GLM. PSTHs shown in (f). (PDF) [file pcbi.1008768.s001.pdf]

S1 Text for: Nonlinear effects of intrinsic dynamics on temporal encoding in a model of avian auditory cortex

Authors: Christof Fehrman[1], Tyler D Robbins[2], and C Daniel Meliza[1,3,*]

Affiliations: [1] Psychology Department, [2], Cognitive Science Program, [3] Neuroscience Graduate Program, University of Virginia, Charlottesville VA 22904, USA

[*] Corresponding author. Email: cdm8j@virginia.edu

S1 Text. Details of GLM estimates for exemplar tonic and phasic models shown in Figs 4A–4F.

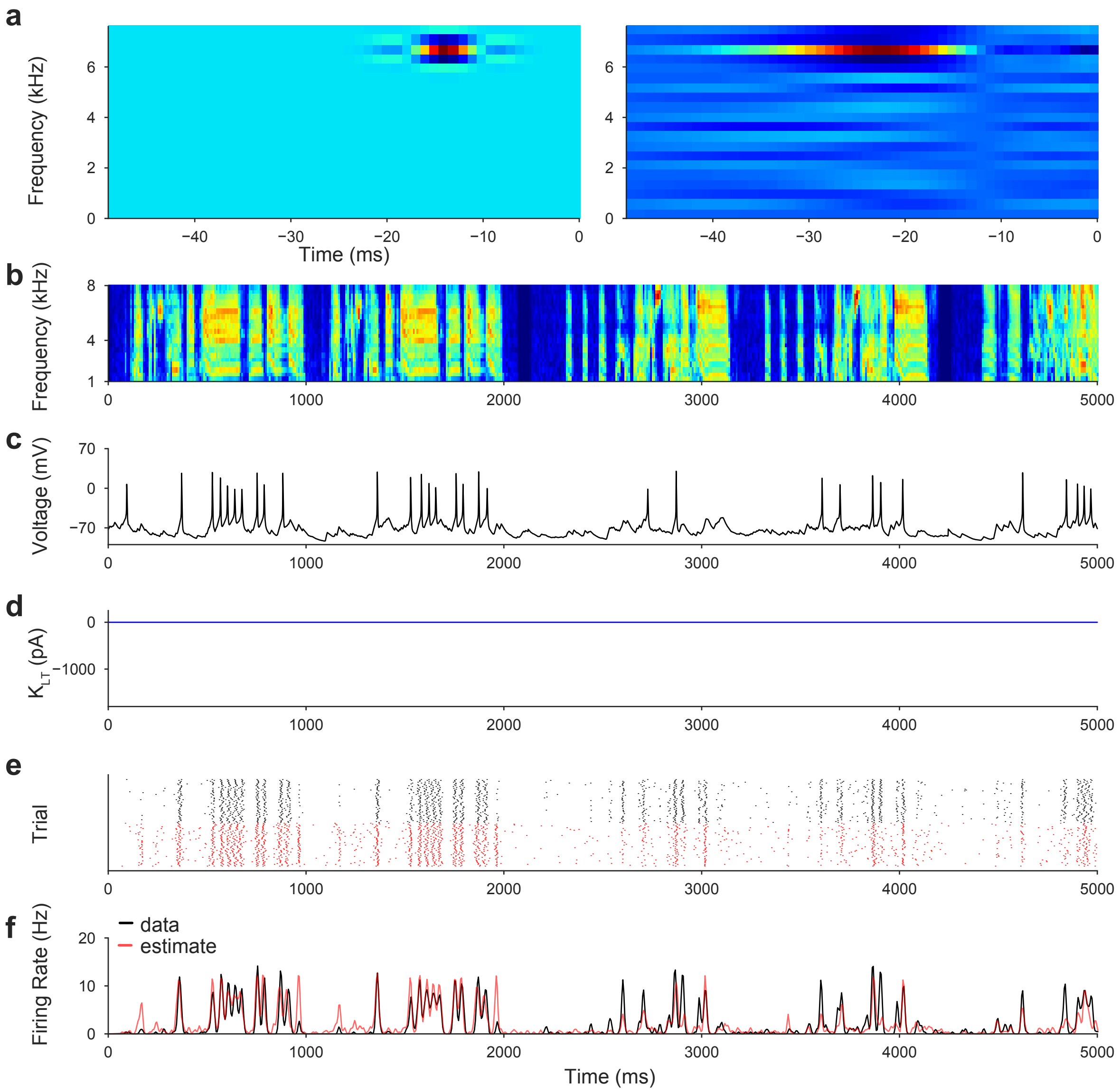

Fig 1. GLM estimate for tonic WB example. Details of GLM estimates for the model in Fig 4A: (a) Left, input RF in the LDC model. Right, estimated RF from GLM. A novel birdsong stimulus was used to compare GLM performance to output of LDC model, with 5s of the spectrogram shown in (b). Voltage traces of LDC model in response to stimulus for a single trial are shown in (c) with corresponding K_{LT} current (d). Spike trains for all 50 trials are shown in (e), with black corresponding to the LDC model and red to the GLM. PSTHs shown in (f).

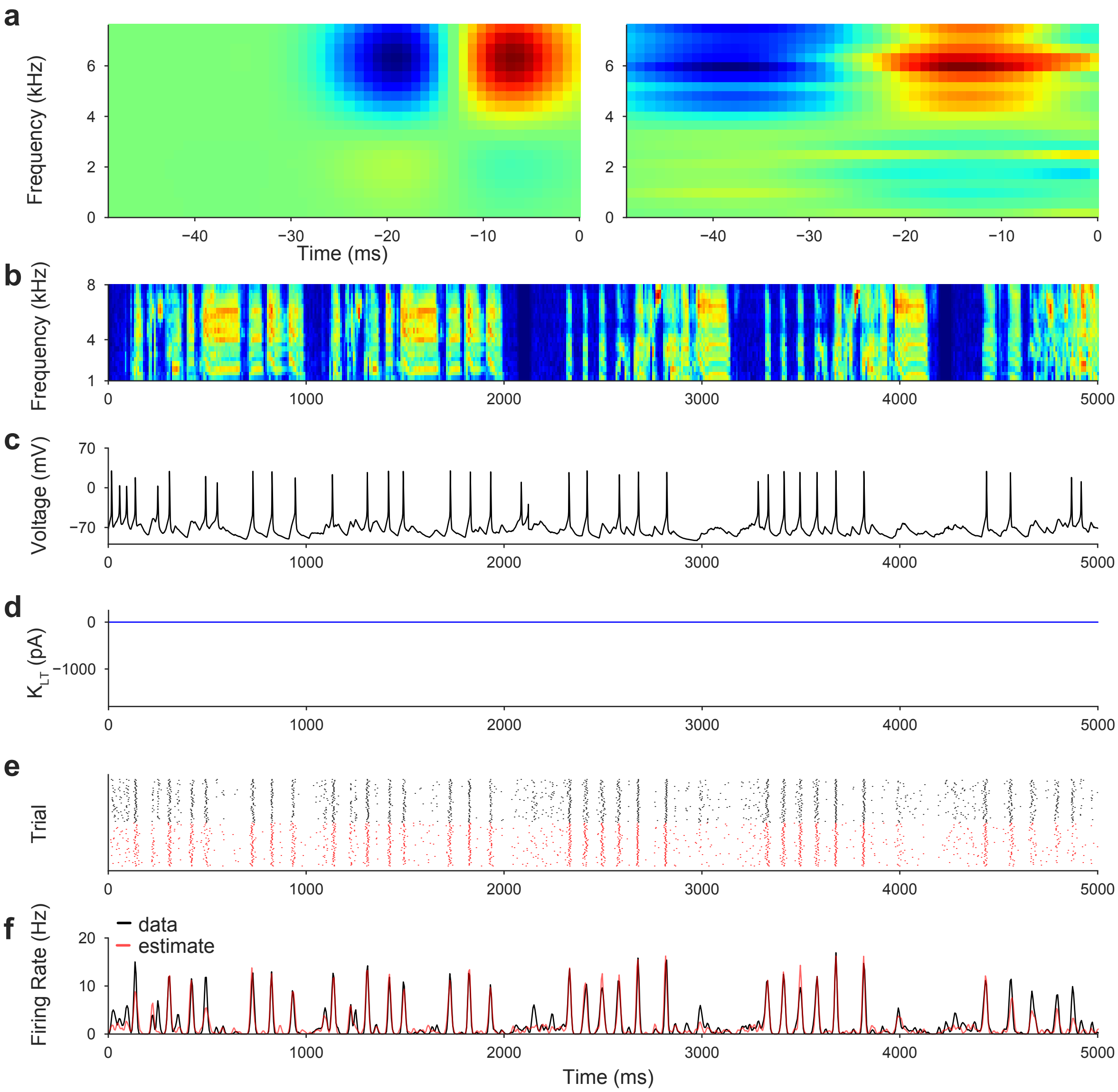

Fig 2. GLM estimate for tonic BP-L example. Details of GLM estimates for the model in Fig 4B; same format as Fig A in S1 Text

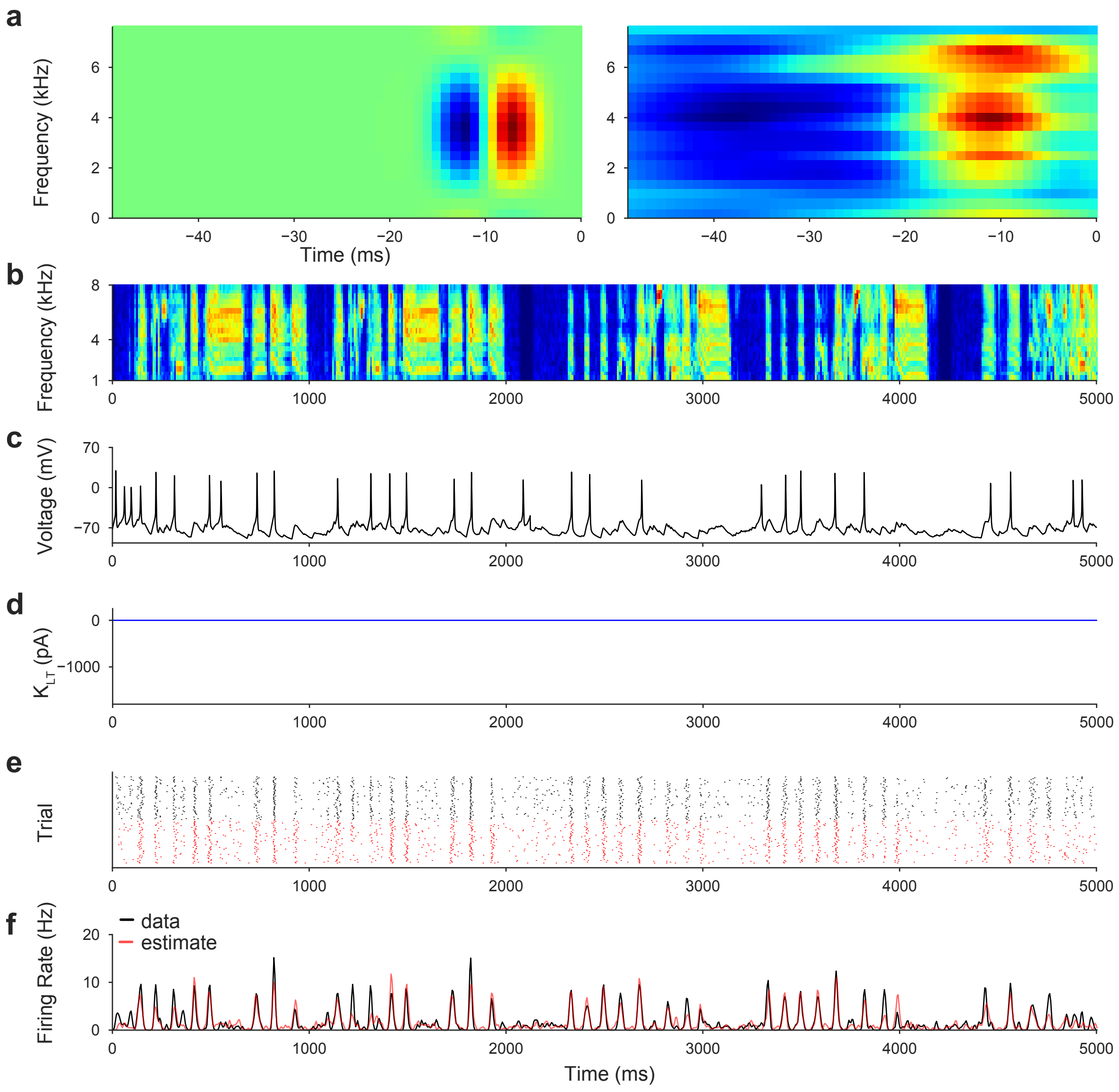

Fig 3. GLM estimate for tonic BP-H example. Details of GLM estimates for the model in Fig 4C; same format as Fig A in S1 Text

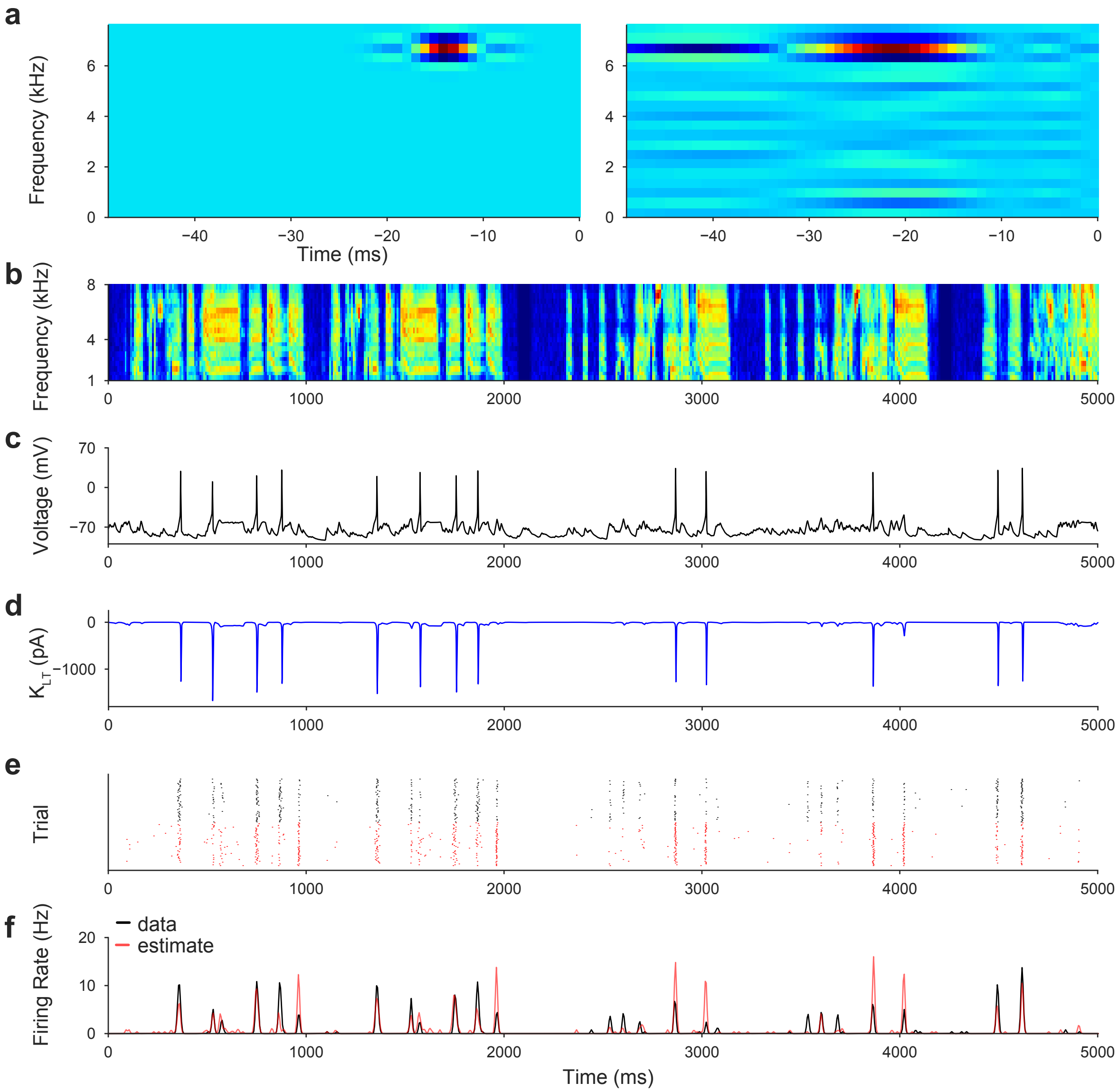

Fig 4. GLM estimate for phasic WB example. Details of GLM estimates for the model in Fig 4D; same format as Fig A in S1 Text

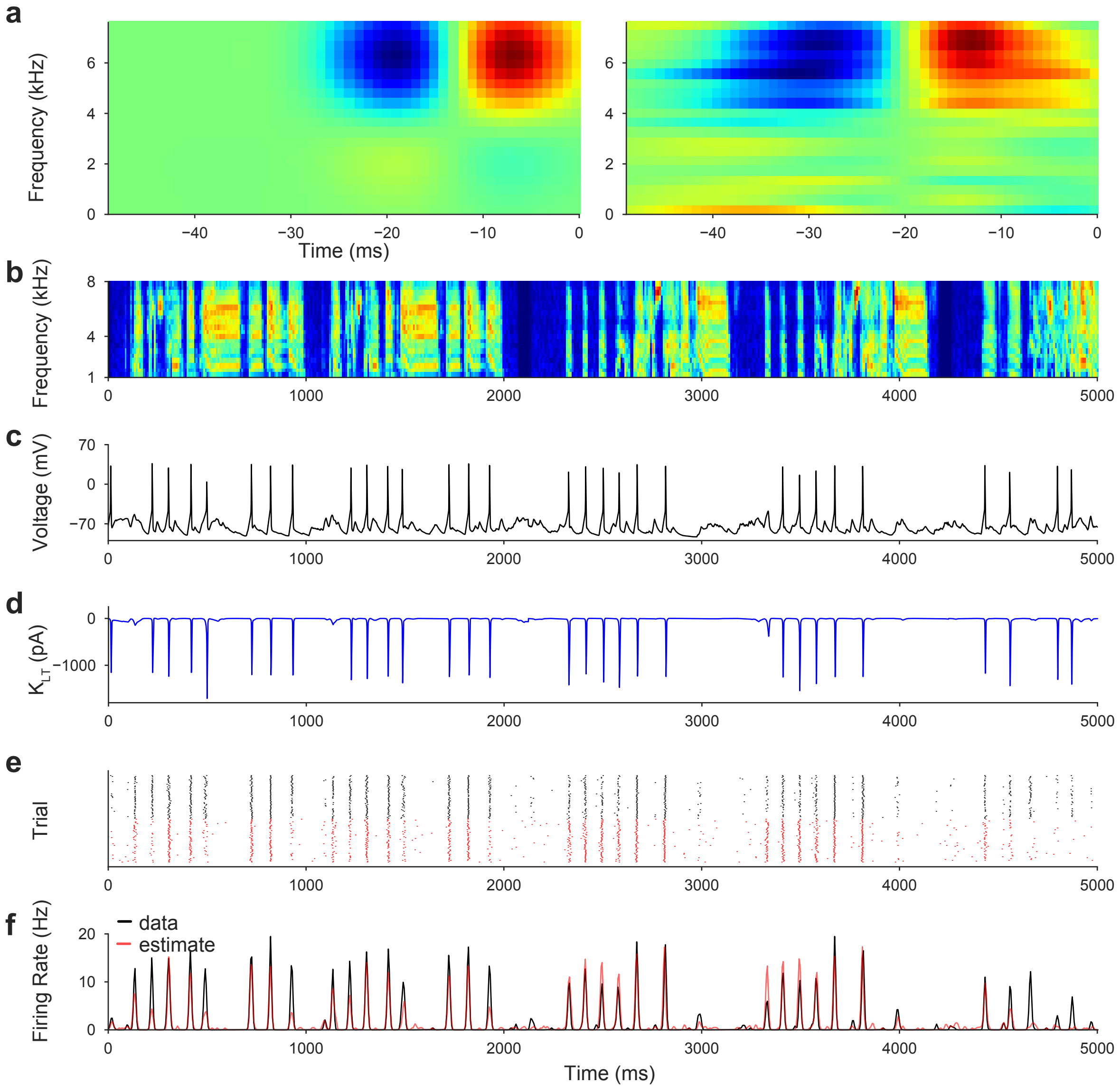

Fig 5. GLM estimate for phasic BP-L example. Details of GLM estimates for the model in Fig 4E; same format as Fig A in S1 Text

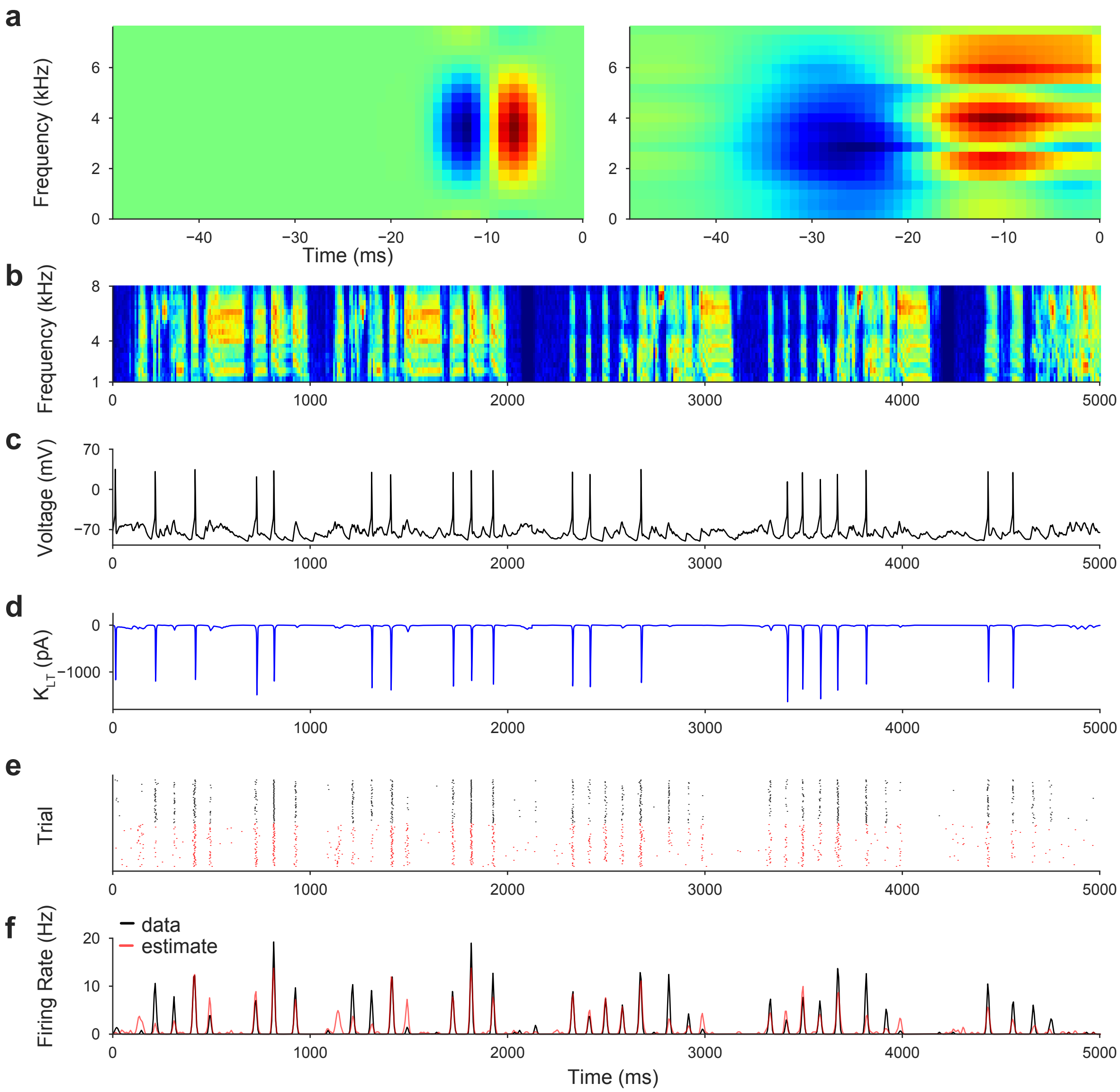

Fig 6. GLM estimate for phasic BP-H example. Details of GLM estimates for the model in Fig 4E; same format as Fig A in S1 Text
